# Controlling schistosomiasis with praziquantel: How much longer without a viable alternative?

**DOI:** 10.1186/s40249-017-0286-2

**Published:** 2017-03-28

**Authors:** Robert Bergquist, Jürg Utzinger, Jennifer Keiser

**Affiliations:** 1 Ingerod, Brastad, Sweden; 2 Swiss Tropical and Public Health Institute, P.O. Box, CH-4002 Basel, Switzerland; 3 University of Basel, P.O. Box, CH-4003 Basel, Switzerland

**Keywords:** Chemotherapy, Drug repositioning, Elimination, Morbidity control, Praziquantel, Schistosomiasis

## Abstract

The current approach of morbidity control of schistosomiasis, a helminth disease of poverty with considerable public health and socioeconomic impact, is based on preventive chemotherapy with praziquantel. There is a pressing need for new drugs against this disease whose control entirely depends on this single drug that has been widely used over the past 40 years. We argue that a broader anthelminthic approach supplementing praziquantel with new antischistosomals targeting different parasite development stages would not only increase efficacy but also reduce the risk for drug resistance. Repositioning drugs already approved for other diseases provides a shortcut to clinical trials, as it is expected that such drugs rapidly pass the regulatory authorities. The antischistosomal properties of antimalarial drugs (e.g., semisynthetic artemisinins, synthetic trioxolanes, trioxaquines and mefloquine) and of drugs being developed or registered for other purposes (e.g., moxidectin and miltefosin), administered alone or in combination with praziquantel, have been tested in the laboratory and clinical trials. Another avenue to follow is the continued search for new antischistosomal properties in plants. Here, we summarise recent progress made in schistosomiasis chemotherapy, placing particular emphasis on repositioning of existing drugs against schistosomiasis.

**Electronic supplementary material:**

The online version of this article (doi:10.1186/s40249-017-0286-2) contains supplementary material, which is available to authorized users.

## Multilingual abstracts

Please see Additional file 1 for translations of the five official working languages of the United Nations.

## Background

Schistosomiasis describes a complex of acute and mainly chronic helminth infection with considerable adverse influence on people’s health and wellbeing [1, 2]. The disease is one of today’s foremost neglected tropical diseases (NTDs) and a disease of poverty [3, 4]. The global burden of schistosomiasis is estimated at 2.6 million disability-adjusted life years (DALYs) [5]. Although the number of healthy days lost annually is far below those calculated for HIV/AIDS, malaria and tuberculosis, the perceived importance of schistosomiasis is now more realistic than 10–15 years ago. Indeed, due to the inclusion of also mild symptoms, such as anaemia, diarrhoea, dysuria and exercise intolerance [6, 7], which were not previously counted by the Global Burden of Disease (GBD) study, schistosomiasis now comes second on the list of 18 (after the intestinal nematodes that are counted together) NTDs put forth by the World Health Organization (WHO) [5, 8].

Schistosomiasis is caused by one of six different species of the trematode worm *Schistosoma*, with the great majority of cases either infected with *Schistosoma haematobium*, *S. japonicum* or *S. mansoni* [2, 9]. The African continent harbours both *S. haematobium* and *S. mansoni* with an estimated 90% of all cases in the world concentrated in sub-Saharan Africa [2, 10]. *S. mansoni* additionally occurs in Latin America, most importantly in Brazil [11], while *S. japonicum* remains endemic in seven provinces of the People’s Republic of China, The Philippines and three small Indonesian foci [12]. *S. guineensis*, *S. intercalatum* and *S. mekongi* are not only in a clear minority, but also geographically limited; the former two along parts of the Congo River and in lower Guinea on the African continent [13], while *S. mekongi* is restricted to small foci around the border between Cambodia and Lao People’s Democratic Republic [14].

The pathology characterising schistosomiasis is primarily the result of immune responses against schistosome eggs trapped in the tissues, but blood loss caused by the adult worm is also an important symptom. Haematuria and dysuria are typical early signs of the urogenital form of the infection (caused by *S. haematobium*) with kidneys and bladder becoming seriously affected in chronic infections [15]. The intestinal form (caused by any of the five other species) results in blood in the stool and diarrhoea with the liver and spleen affected in the longer term [2, 15]. Morbidity has been strongly suppressed as a result of preventive chemotherapy that is the periodic large-scale administration of praziquantel to school-aged children and other high-risk groups without prior diagnosis [16]. A systematic review and geostatistical analysis pertaining to schistosomiasis in sub-Saharan Africa suggests that the prevalence and distribution of the disease has declined in recent years, most likely as a result of preventive chemotherapy, coupled with social and economic development [10]. Of note, in 2015, among the 218.7 million people requiring preventive chemotherapy against schistosomiasis, 65.2 million people were administered praziquantel, owing to a global coverage of 29.8% [17].

Praziquantel, a highly efficacious pyrazinoisoquinoline derivative with a good safety profile and a broad spectrum against helminth infections, remains the drug of choice for schistosomiasis chemotherapy since almost 40 years [18, 19]. Thanks to the good therapeutic and safety index [20], and a donation programme from Merck, praziquantel is the cornerstone in the WHO promoted global strategy against schistosomiasis. Despite reports of strains that have become tolerant or even resistant to praziquantel [21–23], these findings are of little clinical relevance thus far. In the late 1970s/early 1980s, praziquantel replaced the first useful antischistosomal drugs oxamniquine, another quinoline derivative effective against *S. mansoni* [24, 25], and metrifonate, an insecticide based on inhibition of acetyl-cholinesterase that is efficacious against *S. haematobium* [24, 26]. Before that, in the early part of the 1900s (up to around 1975), a number of less efficacious compounds were used in spite of major adverse events, e.g., hycanthone, luchantone, amoscanate, niridazole and oltipraz. Antimony potassium tartrate, a potentially poisonous metalloid without distinctive effect, but known as an emetic and medicine against various ailments since ancient times marked the end of non-specific schistosomiasis chemotherapy [27, 28].

Schistosomiasis control is at a pivotal point in major endemic areas. Elimination has been articulated as an aspiration, but it is clear that it cannot be based on praziquantel alone [29]. The emphasis on preventive chemotherapy campaigns using praziquantel has led to the misconception that there is little need for alternative compounds. Drug discovery run by academic research groups rather than industry has resulted in a variety of new leads, which might serve as starting points for antischistosomal drug discovery. We review progress in research and development of antischistosomal drugs and update the intriguing field how new and old drugs can be utilized in concert against schistosomes in different developmental stages. Our focus is on repositioning of drugs used against diseases other than schistosomiasis, as this strategy might advance existing drugs more rapidly into clinical use for a typical disease of poverty with a deficient market.

## Research and development of antischistosomal drugs

### Setting the scene

The WHO road map for the global control of the NTDs includes schistosomiasis [30]. However, control – let alone elimination – of schistosomiasis has proven difficult because of rapid re-infection due to limited means of control for the intermediate host snail and absence of sanitary facilities in most endemic areas [29, 31, 32]. A variety of approaches, including water, sanitation and hygiene (WASH), information, education and communication (IEC), behavioural change and snail control, have been employed, but preventive chemotherapy with praziquantel has been the fundamental approach since the early 1980s. If, for any reason, this line of attack would falter, there is currently no other remedy to put in its place and the next generation of anthelminthic drugs is yet to appear. Since the last surge of drug discovery for parasitic worm diseases in the 1970s, resulting in the marketing of praziquantel and ivermectin that, together, cure several of the major helminth infections [33], no new drugs for helminthiases have been approved. It is even more worrisome that the drug discovery and development pipeline for NTDs has run dry over the past 40 years [34].

Pre-emptive action is urgently needed to assure that chemotherapy does not dwindle, particularly not in low- and middle-income countries where schistosomiasis inflicts a disproportionately high burden of disease. Judging from the veterinary field, development of drug resistance in helminthiasis can be swift [35] and the situation with respect to malaria is a constant reminder of this risk [36].

### Laboratory studies

The search for new drugs against schistosomiasis has taken various directions, including testing natural compounds, target-based drug discovery or compounds marketed for other indications. Phenotypic screens play an important role in antischistosomal drug discovery [37] and many products of potential value have been found this way.

Natural plant products are an important source of antiparasitic drugs [38, 39]. This is not only potentially fruitful with respect to isolation of new active principles, but would also facilitate the elucidation of their mechanism of action. Herbal extracts have drawn attention due to their broad range of biological activity and the large variety of chemical structures presented. This untapped source of potential anthelminthic compounds remains a novelty and many papers on this topic exist. For example, a search on PubMed on January 23, 2017 using the search terms ‘plant extract’ and ‘schistosomiasis’ yielded 227 publications, while ‘plant extract’ and ‘*Schistosoma*’ yielded 194. It should, however, be noted that not only hits active against schistosomes but also against the snail intermediate host (plant molluscicides) and sometimes against other parasites were discovered [40].

Another strategy is based on using available biologically active natural compounds and their directed chemical modification as recently demonstrated for new triphenylphosphonium derivatives of betulin and betulinic acid [41]. However, rather than discussing the many different schistosomicidal agents discovered in plant extracts here, we refer to recent reviews of the field by de Moraes [42] and Neves and colleagues [43].

The recent sequencing of the genomes of the three key schistosome species has resulted in the discovery of many new possible vaccine and drug targets [44–46]. Moreover, it is conceivable that drug discovery programmes using molecular modelling strategies will increasingly contribute to antischistosomal drug discovery [47, 48].

Work on the antischistosomal properties of various antimalarial drugs, notably semi-synthetic artemisinins, synthetic trioxolanes and mefloquine [49], as well as a few more discussed below, have shown that repurposing of drugs may be the more rapid approach as it provides a shortcut to clinical trials and rapid passage through regulatory authorities. Indeed, the idea of repositioning drugs is an inexpensive, yet effective source of new lead compounds for use against schistosomiasis, which has already resulted in several promising leads [50].

While the antimalarials are the most well-known group of drugs shown to have a strong action against schistosomes in addition to what they are intended for [49] several other pharmaceutical groups also contain compounds with such effect, notably drugs used for cancer therapy [51]. Table 1 contains a list of candidate drugs described in some detail with regard to use against schistosomiasis summarising their effect on adult and juvenile schistosomes, including dosage.

**Table 1:** Activity observed with drug discovery candidates in the *S. mansoni-*mouse model

Chemical compound/drug	Dose	Worm burden reduction (adult)	Worm burden reduction (juvenile)	Reference
Artemether	300 mg/kg/day for 2 days	85.4–98.3%	70%	[52]
Artesunate	150 or 300 mg/kg/day for 7 days	34–49%	67–77%	[53]
Bisquinoline cyclen derivative	400 mg/kg single dose	12.3%	Not done	[74]
Bisquinoline cyclen derivative (Fe^++^ complex)	400 mg/kg single dose	88.4%	Not done	[74]
Bisquinoline cyclen derivative (Mn^++^ complex)	400 mg/kg single dose	74.5%	Not done	[74]
Chlorambucil	2.5 mg/kg/day for 5 days	22.7%	75.8%	[77]
Clofazimine	400 mg/kg single dose	82.7%	Not done	[84]
2,3-dianilinoquinoxaline MMV007224	400 mg/kg single dose	40.8%	Not done	[71]
N,N’-Diarylurea MMV665852	400 mg/kg single dose	52.5%	Not done	[71]
N,N’-Diarylurea MMV665852 analogs	400 mg/kg single dose	9.3–36%	Not done	[72]
Doramectin	10 mg/kg single dose	60.1%	Not done	[84]
Mefloquine	400 mg/kg single dose (adult), 100 mg/kg (juvenile)	77.3%	94.2%	[65]
Mefloquine derivatives	100 mg/kg single dose	0-87%	Not done	[69]
Miltefosin	20 mg/kg daily for 5 days	95.4%	75.5%	[75]
OZ78	200 mg/kg single dose (juvenile), 400 mg/kg single dose (adult)	0%	82%	[57]
OZ209	200 mg/kg single dose (juvenile), 400 mg/kg single dose (adult)	16%	85%	[57]
OZ288	200 mg/kg single dose (juvenile), 400 mg/kg single dose (adult)	52.2%	95.4%	[57]
OZ418	200 mg/kg single dose (juvenile), 400 mg/kg single dose (adult)	80%	100%	[59]
Perhexiline maleate	23, 70 and 400 mg/kg single dose	Not given	Not given	[83]
Piperaquine/OZ277 (Synriam)	40 mg/kg arterolane (OZ277) and 200 mg/kg piperaquine	88.7%	85.4%	[61]
Ferroquine	200 mg/kg and 800 mg/kg single dose	19.4% and 35.6%	Not done	[73]

#### Antimalarials

Natural products have played a major role in drug discovery, in particular for malaria treatment and prophylaxis. While quinine and artemisinin come straight from natural plants, several semi-synthetic agents (e.g., artemether and artesunate) [52, 53] and synthetic derivatives have been at the centre of attention for more than 15 years. Hybrid molecules comprising of a 1,2,4-trioxane domain (as in the artemisinins) linked to amino quinoline (as in chloroquine) were developed in the late 1990s; these trioxaquines thus have a dual mechanism of action, which improves the antimalarial activity over each single compound [54]. Meanwhile, some trioxaquines have also been analysed for their antischistosomal properties with promising results [55], suggesting that the heme molecule is an important target for these compounds [56]. The mechanisms of action of the 1,2,4-trioxolanes remains unknown but is clearly connected with the peroxide bond, which they share with the artemisinins. The compounds OZ78, OZ209 and OZ288 were first studied in *S. mansoni*- and *S. japonicum*-rodent models [57] and several representative compounds with high antischistosomal activity were identified, some of which – such as OZ418 – have improved pharmacokinetic properties [57–60]. The highest activity (worm burden reduction of 72%) was seen after treating hamsters infected with adult *S. mansoni* with OZ288 [57] and mice with OZ418 (worm burden reduction of 86%) [59]. Recently, using rodents infected with *S. mansoni*, Mossallam and colleagues showed high activity of OZ277 combined with piperaquine phosphate, a bisquinoline first synthesised in the 1960s and used extensively in the People’s Republic of China until resistant strains of *Plasmodium falciparum* arose [61]. Bridged 1,2,4,5-tetraoxanes, tricyclic monoperoxides, bridged 1,2,4-trioxolanes, silyl peroxides and hydroxylamines were also studied with the highest antischistosomal activity (in particular in vitro) observed for trioxolanes and tricyclic monoperoxides [62].

Van Nassauw et al. [63] were the first to report an antischistosomal effect by mefloquine, an effective antimalarial agent that was introduced as a successor to chloroquine [64]. They noted that mefloquine significantly reduces egg production in *S. mansoni*-infected mice but they saw no effect on worm burden. Later papers also reported impact on egg maturation but in contrast to this first paper, researchers all noted strong effect with respect to worm burden [65–67]. When different amino ethanols, such as halofantrine, lumefantrin, quinine and mefloquine, were tested in *S. mansoni*-infected mice, mefloquine showed the highest activity [65]. A further important finding is that, in contrast to the effect by artemisinins and praziquantel that target juvenile and adult schistosomes, respectively, all parasite stages are equally affected by mefloquine [65]. Only moderate stereoselectivity was observed testing the activities of the erythro and threo isomers and racemates of mefloquine [66]. In addition, mefloquine had a strong effect on tissue egg load and number of liver granulomas [67]. Additionally, activity has been shown against *S. japonicum* [68] and *S. haematobium* [69]. Moreover, synergistic effects were observed when mefloquine and praziquantel were combined in the animal model [70]. Finally, when evaluating the antischistosomal activities of nine mefloquine-related compounds belonging to the 4-pyridinemethanols, 9-phenanthrenmethanols, and 4-quinolinemethanols, high activity was observed for WR7930 and enpiroline, an antimalarial drug, which had already undergone clinical testing [69].

Another approach is represented by the screening of a library for antischistosomal properties in 200 diverse drug-like and 200 probe-like compounds with confirmed in vitro activity against *P. falciparum* obtained from the Medicines for Malaria Venture (MMV). Thirty-four compounds were found active in in vitro screens, two of which (N,N’-diarylurea and 2,3-dianilinoquinoxaline) identified as early leads after extensive in vitro and in vivo testing; they also had good pharmacokinetic profiles and low cytotoxic potential. In more detail, in vivo experimental work with *S. mansoni* revealed worm burdens reductions of 53% and 41%, respectively, compared to controls [71]. Follow-up studies evaluated the structure-activity relationships of 46 commercially available analogues of MMV665852 larval and adult *S. mansoni* worms in vitro with subsequent in vivo studies. Despite satisfactory in vitro results and *in silico* predictions, only one compound resulted in a statistically significant worm burden reduction (66%) in *S. mansoni*-infected mice. The authors concluded that there is a need to synthesise compounds with improved solubility and pharmacokinetic properties [72].

The ferrocenyl analogue of chloroquine, ferroquine, is an antimalarial in late-stage drug development. However, the drug revealed only moderate in vitro activity against both larval and adult stages of *S. mansoni* and low in vivo activity [73].

Further, a series of synthetic tetra-azamacrocyclic derivatives and their metal complexes, which are effective antimalarial agents were synthesised, characterised and screened in vitro against different schistosomal stages (schistosomula and adult worms). Three compounds (the bisquinoline derivative of cyclen and its Fe^2+^ and Mn^2+^ complexes), highly active in vitro, were selected for in vivo experiments using the *S. mansoni*-mouse model. The worm burden reductions were 12%, 88% and 75%, respectively. The additional fact that the Fe^2+^complex exhibited an activity comparable to that of praziquantel must be deemed a strong indication of a novel drug lead for schistosomiasis [74].

#### Anticancer drugs

It has further been demonstrated that miltefosine, an anticancer alkylphosphocholine, possesses significant activity against different developmental stages of *S. mansoni* [75]. This lead has been further developed using a nanotechnological adjuvant approach based on lipid nanocapsules [76]. The results of this study indicated a strong schistosomicidal effects against both the invasive and the immature stage, further indicated by a significant reduction in worm burdens [76].

Chlorambucil, a nitrogen mustard alkylating agent that has so far mainly been used in the treatment of chronic lymphocytic leukemia, low-grade non-Hodgkin’s lymphoma and Hodgkin’s disease, and is on the WHO list of essential medicines, recently demonstrated an antischistosomal in vitro effect in the form of progressive reductions of worm viability in a dose-dependent manner [77]. In vivo, chlorambucil induced a significant reduction in the total worm (*S. mansoni*) burden, including 89% and 87% intestinal and hepatic egg count reduction, respectively, with the highest in vivo efficacy against the juvenile stage of *S. mansoni* [77].

Cowan et al. studied the approved oncology drug set of the National Cancer Institute’s Developmental Therapeutic Program against *S. mansoni* in vitro and in vivo. Of six compounds studied in vivo, the highest activity was observed with two kinase inhibitors – trametinib and vandetanib with respective worm burden reductions of 63.6% and 48.1% – after a single oral dose of 400 mg/kg [78]. In schistosomes, conserved protein kinases possess pivotal roles contributing not only to reproduction processes, but also to the pathology of schistosomiasis [79]. Kinases represent therefore attractive targets for new-generation antischistosomal drugs.

#### Miscellaneous products

The aryl hydantoin Ro 13–3978 is a compound discovered by Hoffmann-La Roche more than two decades ago with good in vivo antischistosomal activity [80]. Ro 13–3978 is structurally similar to the antiandrogenic drug nilutamide, which has moderate antischistosomal properties [81]. To reduce the antiandrogenic effect, Wang and colleagues designed a number of analogs incorporating substructures and functional groups and identified several compounds with high antischistosomal efficacy that were less antiandrogenic [82].

Other unrelated drugs have also received interest. For example, a recent report has shown that perhexiline maleate, a drug used for heartburn (angina) by improving myocardial oxygen utilization, surprisingly reacted when a library with an ATP-based luminescent assay based on *S. mansoni* schistosomula was screened [83]. This drug was found to exhibit a marked lethal effect on all *S. mansoni* parasite life stages in vivo resulting in tegumental damage in the adult male worm and impaired egg production in the female worm [83]. Although the preliminary in vivo experiments only resulted in moderate egg reductions, this drug warrants deeper investigation and if found as useful clinically as the in vivo experiments indicate (Table 1), the road to registration should be short.

In another scheme, researchers found 121 compounds active against *S. mansoni* schistosomula out of 1 600 compounds approved by United States’ Food and Drug Administration (FDA). When advancing to an adult worm screen, the list shrank by more than 70% and when available pharmacokinetic and toxicity data for these compounds were considered, only 11 remained, two of which (i.e., doramectin and clofazimine) resulted in worm burden reductions of 60% and 83%, respectively, when tested in vivo against *S. mansoni* [84]. These two active drugs are widely available and constitute excellent starting points for developing novel drug classes against schistosomiasis.

Similarly, Abdulla et al. [85] tested a large number of natural and synthetic compounds against *S. mansoni* schistosomula, many of which were approved drugs to find starting points for developing new leads. The various compounds identified have been posted online as a community resource.

### Clinical studies

Drug discovery and follow-up development is an expensive endeavour. Repositioning of drugs already approved for human use offers a way out of this impasse, at least from the part of the studies concerned with safety. Phase II/IV clinical trials are still needed to make sure that the drugs work as planned with respect to the pathogens they are supposed to protect against. In an endeavour to find drugs against schistosomiasis, antimalarials were the most widely studied drugs, as summarised in Table 2.

**Table 2:** Summary of four recent clinical trials with drugs repositioned for use against chronic infections with *S. mansoni* and *S. haematobium*

Compound	*S. mansoni*		*S. haematobium*		Reference
	Cure rate (%)	Egg reduction rate (%)	Cure rate (%)	Egg reduction rate (%)	
Artesunate	Not done	Not done	25	85	[104]
Mefloquine	Not done	Not done	21	74	[104]
Mefloquine-artesunate	Not done	Not done	61	96	[104]
Mefloquine-artesunate-praziquantel	Not done	Not done	29	96	[105]
Moxidectin	13	71	15	9	[103]
Synriam	7	65	11	0	[103]
Praziquantel-Synriam	27	78	60	96	[103]
ARA Arachidonic acid	13–50	0–64	Not done	Not done	[109]

The artemisinins have been discussed extensively in previous reviews [49, 50, 58, 86–91]. Briefly, leaf extracts from the plant *Artemisia annua* recommended in Chinese traditional medicine against malaria (and other ailments), constituted the starting point for the development of the artemisinin-based combination therapy (ACT) currently in use against this infection [92, 93]. The expansion of the therapeutic effect of the artemisinins from *Artemisia* leaves to include *S. japonicum* schistosomes discovered in the early 1980s was completely unexpected [94]. This effect by the semi-synthetic artemisinin derivative artemether was later also confirmed for *S. mansoni* [52] and *S. haematobium* [95] in experimental animals. Subsequent clinical trials confirmed the protective efficacy for artemether [96, 97] and artesunate [98] and encouraged many articles discussing the advantage (and potential disadvantages) of including the artemisinins along with praziquantel in areas endemic for schistosomiasis and co-endemic for malaria [88, 90].

In more detail, the antischistosomal effect of the artemisinins is primarily directed against juvenile schistosomes, while that of praziquantel works on the very day of infection, after which the parasite does not become susceptible against this drug again until it reaches adulthood 4–5 weeks later [87]. Although the periods of susceptibility vary slightly between the different schistosome species infective to man, the principle of age-correlated impact remains the same. Surprisingly, but advantageously, the drugs thus complement each other [87]. Currently, preventive chemotherapy is based on the administration of praziquantel only, which means that only adult worms are killed and that the person treated will be fully repopulated from surviving schistosomula about a month later. Combination therapy would improve the situation but it must also be iterated for as long as the patient lives in an endemic area if precautions against reinfection are not taken (which is difficult and costly in practice). The risk for drug resistance due to widespread use of monotherapy with artemether or artesunate, however, must be taken seriously as the emergence of malaria parasites resistant to the artemisinins have shown [99, 100]. However, used in non-malarious areas, this risk should be limited.

There is no single prophylactic drug that can reliably protect against schistosome infection, but the combination of praziquantel and artemether comes close [101]. On the other hand, the use of this kind of prophylaxis can only be recommended in very special cases; for example during flood relief work in the People’s Republic of China and perhaps when the final steps of elimination of the infection are taken.

Arterolane maleate (OZ277) is one of the first fully synthetic non-artemisinin antimalarial compounds marketed, hence offering a treatment alternative to the artemisinins (Table 2). The arterolane maleate/piperaquine combination, licensed in 2011 as an antimalarial drug under the name of Synriam® [102], has been investigated for activity against chronic schistosome infections in humans. Two single-blind, randomised exploratory Phase II trials have been carried out in adolescents infected with *S. mansoni* and *S. haematobium* in Côte d’Ivoire with cure rate (CR) and egg reduction rate (ERR) based on geometric mean and safety as endpoints. Synriam® showed low efficacy against *S. haematobium* with CR and ERR of 11% and 0%, respectively. Against *S. mansoni* a CR of 7% and an ERR of 64.9% was observed [103]. Despite the low efficacies observed it might be worthwhile to assess the efficacy of Synriam against juvenile schistosome infections.

A few years ago the efficacy and safety of mefloquine (25 mg/kg) was studied in a randomised, exploratory open-label trial against *S. haematobium*. The other treatment arms consisted of artesunate (3 doses of 4 mg/kg), mefloquine-artesunate (3 doses of 100 mg artesunate plus 250 mg mefloquine) and praziquantel (40 mg/kg) alone. CRs of mefloquine, artesunate, mefloquine-artesunate and praziquantel alone against *S. haematobium* at day 26 after treatment were 21%, 25%, 61% and 88%, respectively. Both mefloquine-artesunate and praziquantel alone revealed ERRs in excess of 95% [104]. Based on the aforementioned report documenting synergistic effects when mefloquine and praziquantel were combined [70], a thorough study investigating combination therapy for *S. haematobium* infection in human subjects was initiated. When praziquantel-mefloquine and praziquantel-mefloquine-artesunate combinations were used, the investigators could not find any increased efficacy compared to praziquantel alone (CRs from 26% to 33%), but they observed a higher level of side effects with the mefloquine combinations [105].

Moxidectin, a semisynthetic derivative of nemadectin produced by fermentation by *Streptomyces cyanogriseus* originally developed for the prevention and control of *Dirofilaria* immitis (heartworm) and intestinal worms in dogs [106], is being assessed as an alternative to ivermectin in the treatment of onchocerciasis in humans. In the frame of this programme its effect on concomitant helminths revealed CR and ERR of 64% and 66%, respectively in *S. mansoni*-infected patients [107]. The aforementioned study by Barda and colleagues recently demonstrated low efficacy of moxidectin against *S. haematobium* and low CR and moderate ERR against *S. mansoni* [103].

Arachidonic acid (ARA), which is a normal constituent of cell plasma membranes was studied in schoolchildren with light, moderate or heavy *S. mansoni* infection given a good performance in a proof-of-concept study in children with light infections [108]. ARA (10 mg/kg per day for 15 days) revealed moderate CRs (50% and 60%, respectively) against light infection and low CRs (21% and 20%, respectively) against heavy infection. Praziquantel and ARA combined elicited 83% and 78% CRs in children with light and heavy infection, respectively [109].

## Discussion

Praziquantel is currently the only fully effective antischistosomal drug in use [25]. It has been used in preventive chemotherapy programmes in many endemic countries, in some continuously since more than 15 years [25, 110, 111]. However, it is not until recently that preschool-aged children and pregnant women were included into the recommended target population for preventive chemotherapy [112, 113]. Both these groups are now recommended to be treated with praziquantel when needed; still there are existing gaps due to drug shortage and lack of a pediatric praziquantel formulation [112]. This expanded access to praziquantel, including biannual treatment schedules [114] and use of the drug for the treatment of domestic animals in the context of zoonotic schistosomiasis [115], increases the risk for development of resistance and demands augmented monitoring of drug failure and resistance.

Drug discovery is indeed essential, since the loss of praziquantel due to resistance would not only jeopardize the success achieved, but also close the window of opportunity for assembling an integrated approach, e.g., combining effective chemotherapy with a different approach with a longer reach, e.g., a vaccine [116]. This aspiration rests, however, on fragile grounds as inadequate funding has held back vaccine development and even if some good advances have been made [117], we are still years from potential field application of a schistosomiasis vaccine. In addition, new drugs are an unconditional necessity not only to safeguard the efficacy of chemotherapy, but also because an effect against both juvenile and adult schistosomes, would also affect transmission allowing longer intervals between preventive chemotherapy rounds.

Identification of the antischistosomal activity of the artemisinins constituted the impetus for investigating the properties of natural products with the aim of developing new treatments for various parasitic diseases. Although this progress represents a landmark in the advance towards completely new drugs against schistosomiasis (and also malaria), ongoing research on repositioning existing drugs has already resulted in a wide range of promising, antischistosomal drug candidates. Although no drug candidate with as good efficacy as that of praziquantel has appeared, many drugs show profiles and efficacies offering the prospect of together covering the whole spectrum from 1-day-old schistosomula up to adult worms such as the praziquantel-artemisinin combinations. Even drugs that have the same effect on schistosome worms can complement each other as long as their mechanisms of action differ.

Apart from the advances made with repositioning drugs, further development of trioxolanes and trioxaquines, as well investigation of plant extracts and screening of libraries, deserve further scientific inquiry. Continuing in these footsteps, we recommend establishing an international research programme for prospecting synthetic, semi-synthetic and bioactive compounds from natural products, including plant molluscicides. In vitro and in vivo evaluation of chemical constituents belonging to different classes of chemical compounds should obviously be part of this effort, as should structural determination using genomic and proteomic approaches.

## Conclusions

Summarising the progress in the field of antischistosomal drug discovery, the sad reality is that in 15 years’ time we will likely have no alternatives to praziquantel available. There is a pressing need to strengthen antischistosomal drug discovery, which includes improving the translational science in academia, intensify collaborative approaches, including engagement with the pharmaceutical industry and persuade donors and policy makers that alternatives to praziquantel are necessary for sustaining the control of schistosomiasis and approaching the goal of eliminating this scourge from mankind.

Research into how drugs act at the fundamental level is an important research area as this has a high probability of discovering superior formulations and combinations. Pursuing this approach, the following axes of research should be particularly rewarding:

1. profiling existing drugs and drug candidates with the aim of finding leads to superior derivatives;
2. investigation of different and more efficient combinations of existing drugs;
3. exploration of disparity between host and parasite metabolic pathways;
4. analysis of genomes and transcriptomes with the aim of identifying new drug targets; and
5. monitoring antischistosomal effects in areas where ACTs are used as first-line drugs against malaria.

## Additional file

Additional file 1: Multilingual abstracts in the five official working languages of the United Nations. (PDF 466 kb)
