# Supplementary material for: Controlling schistosomiasis with praziquantel: How much longer without a viable alternative?
Source: Infect Dis Poverty. 2017 Mar 28;6:74. doi: 10.1186/s40249-017-0286-2 (PMC5371198; doi:10.1186/s40249-017-0286-2)
Supplement: Additional file 1: — Multilingual abstracts in the five official working languages of the United Nations. (PDF 466 kb) [file 40249_2017_286_MOESM1_ESM.pdf]

Translation of the abstract into the five official working languages of the United Nations

السيطرة على مرض البلهارسيا مع باستخدام البرازيكوانتل: كم سننتظر لإيجاد بديل قابل للاستخدام؟

روبرت بيرجكويست، يورج أوتسينجر، جنيفر كايزر

الملخص
النهج الحالي لمكافحة المراضة في البلهارسيا، وهو مرض ينتج من الديدان الطفيلية في الأمكان الفقيرة ويؤثر بشدة على الصحة العامة وعلى الوضع الاجتماعي والاقتصادي، وعلاجه الحالي عو العلاج الكيميائي الوقائي باستخدام البرازيكوانتل. هناك حاجة ملحة لعقاقير جديدة ضد هذا المرض الذي يعتمد كليا على هذا دواء وحده والذي استخدم على نطاق واسع لنحو 40 عاما لمكافحة المرض. نحن نرى أن اتباع نهج أوسع في مقاومة الديدان باستبدال أو مكاملة البرازيكوانتل بمضادات للبلهارسيا جديدة تستهدف مراحل تطور الطفيليات المختلفة من شأنه أن يزيد فعالية فحسب، بل يقلل أيضا من خطر مقاومة الأدوية. الأدوية المعاد النظر في وضعها والتي تمت الموافقة عليها بالفعل لأمراض أخرى توفر اختصارا للتجارب السريرية، حيث من المتوقع أن مثل هذه الأدوية يسرع إجازتها بواسطة السلطات التنظيمية. العمل مع التركيز على خصائص مضاد البلهارسيات في مختلف الأدوية المضادة للملاريا (على سبيل المثال مادة الأرتيميسينين شبه صناعية، الترايوكسولان الاصطناعية، الترايوكساكين، المفلوكين) وهناك العديد من الأدوية المسجلة لأغراض أخرى (على سبيل المثال موكسيدكتين، السينريام، الميلتفوزين)، تعطى وحدها أو بالاشتراك مع البرازيكوانتل، تم اختبارها. وسيلة أخرى لمتابعة هي استمرار البحث عن خصائص مضادات بلهارسيا جديدة في النباتات. هنا، نلخص التقدم المحرز مؤخرا في العلاج الكيميائي للبلهارسيا، مع التركيز بشكل خاص على إعادة تنظيم الأدوية الموجودة ضد البلهارسيا.

Translated from English version into Arabic by Mahmoud Sami, through

TRANSLATORS
WITHOUT BORDERS

若无可行替代药品，吡喹酮还能控制血吸虫病多久？

Robert Bergquist, Jürg Utzinger, Jennifer Keiser

摘要
血吸虫病是一种对公共卫生和社会经济具有相当大影响的蠕虫病。目前，控制其发病率的方法是基于吡喹酮的预防性化疗。而它已被广泛使用约 40 年，因此急需新药物控制血吸虫病。我们认为更多的驱虫方法替换或补充吡喹酮以针对不同寄生虫发育阶段的新抗血吸虫药不仅会增加疗效，而且降低耐药性的风险。已批准用于其他疾病的重新定位药物提供了通向临床试验的捷径，因为预计这些药物会迅速通过监管机构的认证。针对各种抗疟药物的抗血吸虫性（如半合成的青蒿素、合成的三氧杂环戊烷、三氧杂喹啉和甲氟喹）和为其他目的注册的各种药物（例如莫昔克丁、synriam 和米替福新）单独或与吡喹酮联合施用的研究工作已经完成测试。另外，继续在植物中寻找抗血吸虫物质。本文总结了血吸虫病化疗最近取得的进展，特别强调了重新定位现有抗血吸虫病的药物。

Translated from English version into Chinese by Jin Chen, edited by Pin Yang

CDC
CHINESE CENTER FOR DISEASE CONTROL AND PREVENTION
NATIONAL INSTITUTE OF PARASITIC DISEASES

Traiter la schistosomiase (bilharziose) par le praziquantel: combien de temps avant une solution viable ?

Robert Bergquist, Jürg Utzinger, Jennifer Keiser

Résumé
La méthode actuelle du "contrôle de la morbidité" due à la schistosomiase (bilharziose), une affection causée par les vers (vermineuse) et associée à la pauvreté avec un impact socio-économique et de santé publique considérable, repose sur une chimiothérapie préventive contenant du praziquantel. Il y a un besoin urgent de nouveaux remèdes pour lutter contre cette affection dont le contrôle dépend exclusivement de ce seul médicament qui a été largement administré depuis environ 40 ans. Nous estimons qu'une approche plus élargie aux antihelminthiques, remplaçant ou complétant le praziquantel avec de nouveaux médicaments contre la schistosomiase ciblant différentes étapes de cycle de vie d'un parasite, aurait non seulement une action plus efficace mais réduirait aussi le risque de résistance clinique aux médicaments. Le repositionnement de médicaments déjà approuvés pour d'autres maladies fournissent un raccourci pour les essais cliniques puisque l'on s'attend à ce que ces médicaments soient rapidement homologués par les autorités réglementaires. Les

travaux se concentrant sur les propriétés des anti- schistosomes de divers médicaments antipaludiques (artémisinines hémi synthétiques, trioxolanes synthétiques, trioxaquines, méfloquines etc) et divers médicaments homologués à d'autres fins (moxidectine, synriam et Miltéfosine etc), administré seul ou en combinaison avec le praziquantel, ont été examinés. Une autre voie à suivre serait de poursuivre les recherches des nouvelles propriétés des anti schistosomes dans les végétaux. Nous résumons ici les récents progrès réalisés dans le traitement de la schistosomiase par la chimiothérapie en insistant particulièrement sur le repositionnement de médicaments existants contre la schistosomiase.

Translated from English version into French by veromarie, through

TRANSLATORS
WITHOUT BORDERS

Контроль за шистосомозом с помощью празиквантела: как долго без жизнеспособной альтернативы?

Robert Bergquist, Jürg Utzinger, Jennifer Keiser

Краткий обзор

Современный подход к контролю над заболеваемостью шистосомозом, являющимся гельминтным заболеванием, связанным с бедностью и имеющим значительное влияние на общественное здравоохранение и экономику, основан на лечении профилактической химиотерапией с использованием празиквантела. Стала очевидной срочная необходимость в новых лекарственных препаратах против этой болезни, контроль над которой в данный момент полностью зависит от одного лекарства, широко используемого на протяжении почти 40 лет. Мы считаем, что более широкий антигельминтный подход, заменяющий или дополняющий празиквантел новыми противошистосомозными препаратами, нацеленными на различные стадии развития паразитов, не только бы способствовал увеличению эффективности лечения, но и также снижению риска лекарственной устойчивости. Перепрофилированные лекарства, ранее одобренные для лечения других болезней, предполагают быстрый переход к клиническим испытаниям, так как ожидается, что подобные лекарства получат одобрение регулирующих органов в самые короткие сроки. Были проведены тесты противошистосомозных свойств у противомалярийных препаратов (например, полусинтетические артемизины, синтетические триоксоланы, мефлохин и trioxaquines), а также у препаратов, зарегистрированных для лечения других заболеваний (например, моксидектин, милтефозин и synriam), принимаемых отдельно или совместно с празиквантелом. Другое направление – это продолжающиеся поиски противошистосомозных свойств растений. Здесь мы подводим итог последних достижений в лечении шистосомоза химиотерапией, делая особенный акцент на перепрофилирование уже существующих лекарств для лечения этого заболевания.

Translated from English version into Russian by Dmitry Esin

El control de la esquistosomiasis con praziquantel: ¿cuánto tiempo se puede seguir sin una alternativa viable?

Robert Bergquist, Jürg Utzinger, Jennifer Keiser

Resumen

El enfoque actual para el control de la morbilidad de la esquistosomiasis, una enfermedad helmíntica de la pobreza que tiene serias repercusiones a nivel socioeconómico y sobre la salud pública, se basa en la quimioterapia preventiva con praziquantel. Existe la necesidad urgente de producir nuevos fármacos contra esta enfermedad, cuyo control depende íntegramente de un único fármaco que se ha utilizado ampliamente por casi 40 años. Nuestro argumento es que un enfoque antihelmíntico más amplio, que reemplace o complemente al praziquantel con nuevos fármacos que se focalizen en diferentes etapas de desarrollo del parásito, no sólo aumentaría la eficacia, sino también reduciría el riesgo de resistencia a los medicamentos. Los fármacos reposicionados, ya aprobados para otras enfermedades, brindan acceso directo a los ensayos clínicos, dado que se espera que estas drogas sean aprobadas rápidamente por las autoridades reguladoras. Se han probado trabajos centrados en las propiedades de diversos fármacos antipalúdicos para tratar la esquistosomiasis (por ejemplo, artemisininas semisintéticas, trioxolanos sintéticos, trioxaquines y mefloquina), y diversos medicamentos registrados para otros fines (por ejemplo, moxidectina, synriam y miltefosina), administrados solos o en

combinación con praziquantel. Otro camino a seguir es continuar buscando en las plantas nuevas propiedades para tratar la esquistosomiasis. El presente es un resumen de los últimos avances en materia del uso de la quimioterapia contra la esquistosomiasis, con especial énfasis en el reposicionamiento de los fármacos existentes contra la enfermedad.

Translated from English version into Spanish by Maria Paula Gorgone, through

TRANSLATORS
WITHOUT BORDERS
